# State-of-the-Art Dermatophyte Infections: Epidemiology Aspects, Pathophysiology, and Resistance Mechanisms

**DOI:** 10.3390/jof7080629

**Published:** 2021-08-03

**Authors:** Nilce M. Martinez-Rossi, Nalu T. A. Peres, Tamires A. Bitencourt, Maíra P. Martins, Antonio Rossi

**Affiliations:** 1 Department of Genetics, Ribeirão Preto Medical School, University of São Paulo, USP, Ribeirão Preto 14049-900, SP, Brazil; naluperes@gmail.com (N.T.A.P.); tabitencourt@yahoo.com.br (T.A.B.); mairapompeu@hotmail.com (M.P.M.); anrossi@usp.br (A.R.); 2 Department of Microbiology, Institute of Biological Sciences, Federal University of Minas Gerais, Belo Horizonte 31270-901, MG, Brazil

**Keywords:** *Trichophyton*, dermatophytosis, cutaneous infection, fungal-host interaction, virulence traits, transcription factor, resistance mechanisms, keratin degradation

## Abstract

The burden of fungal infections is not widely appreciated. Although these infections are responsible for over one million deaths annually, it is estimated that one billion people are affected by severe fungal diseases. Mycoses of nails and skin, primarily caused by fungi known as dermatophytes, are the most common fungal infections. *Trichophyton rubrum* appears to be the most common causative agent of dermatophytosis, followed by *Trichophyton interdigitale.* An estimated 25% of the world’s population suffers from dermatomycosis. Although these infections are not lethal, they compromise the quality of life of infected patients. The outcome of antidermatophytic treatments is impaired by various conditions, such as resistance and tolerance of certain dermatophyte strains. The adage “know your enemy” must be the focus of fungal research. There is an urgent need to increase awareness about the significance of these infections with precise epidemiological data and to improve knowledge regarding fungal biology and pathogenesis, with an emphasis on adaptive mechanisms to tackle adverse conditions from host counteractions. This review outlines the current knowledge about dermatophyte infections, with a focus on signaling pathways required for fungal infection establishment and a broad perspective on cellular and molecular factors involved in antifungal resistance and tolerance.

## 1. Introduction

A dermatomycosis is the most frequent form of mycoses, which includes infections of the skin, nail, and hair caused by yeasts and filamentous fungi, and affects approximately 25% of the world’s population. When the etiologic agent is identified as a keratinolytic filamentous fungus classified as a dermatophyte, the infection is diagnosed as dermatophytosis, also known as tinea or ringworm. These fungi are cosmopolitan pathogens encountered in many ecological niches, such as soil, as well as human and animal keratinous tissues. Geophilic species found in soil are saprophytic and rarely cause infections in humans and animals. In contrast, zoophilic dermatophytes are mainly found as pathogens in animals and occasionally in humans. These species may be carried by animals in their fur and may cause asymptomatic or symptomatic acute and highly inflammatory infections in both animals and humans. Anthropophilic species are highly adapted to human hosts, transmitted mainly by indirect contact through the scales in the environment/clothes and direct contact between humans, triggering a mild inflammatory response and chronic infection [1].

Dermatophytosis has a significant impact on the patients’ quality of life by affecting psychological, economic, and social aspects, and are associated with anxiety, depression, and low self-esteem, mainly due to discomfort regarding pruritus and cosmetic issues [2,3]. Clinical manifestations include circular, erythematous, and scaly lesions on the skin, while nail infections (tinea unguium or onychomycosis) lead to discoloration, thickening, desquamation, and separation from the nail bed [4]. Atypical manifestations have also been reported, misguiding the clinical diagnosis due to similarities to other cutaneous diseases, such as eczema, psoriasis, impetigo, seborrheic dermatitis, erythema multiforme, lupus erythematosus, dermatitis herpetiformis, polymorphous light eruption, and rosacea [5,6]. Majocchi’s granuloma is a rare manifestation, mostly caused by the anthropophilic dermatophyte *Trichophyton rubrum* or by non-dermatophyte filamentous fungi such as *Aspergillus* spp. It affects immunocompetent patients and is characterized as nodular perifollicular granuloma with the infiltration of macrophages, neutrophils, epithelioid cells, multinucleated giant cells, and lymphoid cells [7]. Although often associated with cutaneous infections, dermatophytosis may become invasive, especially in immunocompromised and predisposed patients, and may present as nodular, ulcerative lesions and abscesses [5]. Underlying conditions associated with deep dermatophytosis include solid organ transplantation, HIV infection, use of immunosuppressive drugs, such as systemic corticosteroids, and immunodeficiencies [8]. Moreover, cases of tinea faciei (or mask tinea) have been reported in India, associated with the increased use of face masks to avoid contamination during the recent COVID-19 pandemic caused by SARS-CoV-2 [9].

Among the factors that increase predisposition to dermatophytosis are environmental factors, such as humidity and temperature, pathogens’ virulence traits and hosts’ immunological and health status, genetics, occupation, hygiene, contact with animals, and socioeconomic factors [10]. Genetic factors have been associated with onychomycosis; HLA-DR4 is associated with protection and HLA-DR8 and HLA-DR1 with susceptibility [11]. In addition, invasive and disseminated dermatophytosis have been associated with *CARD9* (caspase recruitment domain-containing protein 9) mutations and impairment of the Th17 immune response [12].

Accurate diagnosis is essential to improve the prescription of therapeutic strategies suitable for each case and etiological agent. Diagnosis of dermatophytosis relies on clinical aspects combined with conventional methodologies, such as microscopy and culture, with the aim of evaluating fungal morphology and physiology. Molecular techniques based on sequencing of internal transcribed spacer (ITS) and other genomic regions, polymerase chain reaction (PCR), and matrix-assisted laser desorption/ionization time-of-flight mass spectrometry (MALDI-TOF MS) are rarely used in clinics and are mainly applied in epidemiological and evolutionary studies [13]. However, there is an increasing need for standardization of these methodologies due to the high demand for fungal identification from clinical cases of cutaneous infections in humans and animals. Classically, dermatophytes are grouped into three genera: *Trichophyton, Microsporum,* and *Epidermophyton.* However, a recent study using multilocus sequencing included six more genera: *Arthroderma, Nannizzia, Ctenomyces, Lophophyton, Guarromyces,* and *Paraphyton* [14].

The increased frequency of dermatophytosis is due to several factors, such as the use of occlusive footwear, inadequate hygiene, socioeconomic status, occupation, climate change, predisposing factors (such as immunosuppression/deficiency and diabetes), and improvements in diagnostic tests and medical care [2,10,13]. In recent years, an epidemic-like dermatophytosis scenario has been observed in India. The prevalence of dermatophytosis ranges from 6.09% to 61.5%, depending on the region, with the highest prevalence reported in the North [15]. In a review of cases in India, the authors reported that the most prevalent clinical manifestations were tinea corporis and tinea cruris, with the main causative agents being *Trichophyton mentagrophytes*, *T. rubrum,* and *Trichophyton interdigitale* [15]. Onychomycosis and tinea unguium are common in the elderly. However, it may occur at any age and may also occur concomitantly with tinea pedis and is mainly associated with occlusive footwear and humidity. The most common dermatophytes isolated from these cases were *T. rubrum* and *T. mentagrophytes.* However, yeasts and other filamentous fungi are also causative agents of onychomycosis [16]. Tinea capitis, commonly reported in school-age children, is mostly caused by *Microsporum canis*, *Trichophyton tonsurans*, *Trichophyton violaceum*, *Trichophyton soudanense*, and *Microsporum audouinii* [17,18]. An epidemiological survey of dermatophytosis in Switzerland from 2001 to 2018 revealed that the main etiological agents isolated from cases of tinea pedis and unguium were *T. rubrum* and *T. interdigitale* [19]. *Trichophyton violaceum, T. soudanense,* and *T. tonsurans* were mainly isolated from patients with tinea capitis. This study also showed that dogs, cats, and guinea pigs are the main reservoirs of zoophilic species.

The occurrence of cutaneous dermatophytosis may also lead to invasive and deep infections. A systematic review of cases of deep dermatophytosis worldwide, from 2000 to 2020 [20], showed that half of the patients presented chronic cutaneous infections at the same anatomical site before invasive lesions. Predisposing factors included the use of immunosuppressants, solid organ transplantation, mutations in the *CARD9* or *STAT3* (signal transducer and activator of transcription 3) genes, diabetes, and trauma. In this study, *T. rubrum* was the most prevalent dermatophyte, isolated in 53.1% of invasive cases, followed by *T. mentagrophytes*, *M. canis*, *T. tonsurans*, *T. interdigitale*, and *T. violaceum*. Other dermatophytes associated with these invasive cases were *Trichophyton schoenleinii*, *Trichophyton verrucosum*, *Nannizzia gypsea* (formerly *Microsporum gypseum*), *Microsporum inferrugineum*, *M. audouinni*, and *Arthroderma gypseum* [20].

Several factors have been described in the pathogenesis of dermatophytosis, such as the secreted enzymes that enable keratin degradation, metabolic changes to use nutrients from the host tissue, adaptation to skin pH, transcription factors that regulate fungal adaptation to stress, and extracellular vesicles that modulate the host immune response [21,22,23,24,25,26]. During infection, dermatophytes trigger the release of cytokines and chemokines that contribute to the inflammatory response in the skin. While zoophilic species induce a broad range of immunological mediators, anthropophilic dermatophytes induce lower levels of these molecules, corroborating the histological and clinical manifestations observed in dermatophytosis [27].

Treatment of dermatophytosis relies on systemic and topical formulations and combinatory therapy aiming at a fungal clearance and decreasing the possibility of acquiring and selecting resistance. Among the most frequently used drugs are terbinafine, itraconazole, fluconazole, and griseofulvin [28]. However, in some European countries, griseofulvin is no longer used [18]. Moreover, new therapeutic strategies, drug targets, and synthetic and natural compounds have been evaluated and proposed to treat these infections, including reassessing the use of undecanoic acid [29,30]. Treatment is long, with some adverse effects, leading to abandonment. Consequently, relapses and recalcitrant infections are often reported. This may also lead to the selection of resistant strains, which are becoming more frequent. Resistance mechanisms in dermatophytes involve the overexpression of transmembrane efflux proteins, mutations in antifungal targets, upregulation of signaling pathways in response to stress, and detoxification enzymes [29,31,32,33,34,35].

Recent advances in dermatophyte infection models, availability of genomic and transcriptomic data, and improvements in genetic manipulation methodologies have significantly impacted studies on the pathogenic and adaptive processes of these pathogens. Given the burden of fungal infections worldwide and the increasing prevalence of dermatophytosis, this review highlights the latest findings on dermatophyte pathogenicity and antifungal resistance and tolerance.

## 2. Virulence Attributes of Dermatophytes

### 2.1. Infection Models and Fungal-Host Interaction

Infection models to study dermatophytosis include in vitro, in vivo, and ex vivo approaches, which, in combination, greatly increase the knowledge about the pathogenesis of dermatophytosis and dermatophyte biology. These studies are pivotal for establishing strategies to control, prevent, and treat infections caused by these filamentous fungi. Overall, in vitro investigations rely on substrates present in the host tissue, such as keratin, protein, and lipids, providing insights into dermatophyte metabolism and nutrient sensing during infection [36,37,38]. Moreover, in vitro interactions with cells, such as keratinocytes, macrophages, neutrophils, and *Acanthamoeba*, have also been used to understand fungal responses to host cells and immune defenses [39,40,41]. In vivo animal models include the use of guinea pigs, mice, and invertebrates (such as *Galleria mellonella* larvae), which have also been useful for evaluating the activity of antifungal drugs [38,42,43,44]. Among the ex vivo methodologies used for dermatophytes are animal and human skin, nail explants, and reconstituted epidermal tissue [25]. Furthermore, each model has its limitations and advantages, and it is useful in providing significant contributions to the field of medical mycology.

The ability of fungi to sense and adapt to the host is crucial for successful infection [45]. Fungal cells colonize host keratinized structures such as nails, hair, and skin in a process that relies upon mechanical and biochemical rearrangements [46]. The attachment of fungal cells to host tissues is mediated by the expression of carbohydrate-specific “adhesins” located on the conidial surface and fibril projections that connect adjacent arthroconidia with the skin [47]. The subtilisin family protease Sub3 is involved in the attachment of *M. canis* to the reconstructed feline epidermis, suggesting that proteases also possess adhesion properties [48]. In addition, the gene that encodes an ortholog of the cell wall protein Sowgp was upregulated in the early stages of *T. rubrum* infection, reinforcing its role in virulence [25]. Tandem repeats containing proteins are potential candidates for cell-to-cell junctions, surface adhesion, and fungal virulence [49]. Recently, genes that encode cell wall proteins with tandem repeats have been investigated, revealing the upregulation of *mad1 adhesin* (TERG_08771), *msb2* (TERG_05644), *scw11* (TERG_05576), and *mps1* (TERG_08369) during *T. rubrum* growth on keratin substrates [50]. In silico characterization of the protein codified by TERG_08771 (Mad 1 adhesin), demonstrated the presence of domains potentially involved in adhesion, such as collagen triple helix repeat, mucin-like glycoprotein, and flocculin type 3 repeat [51]. In addition, gene expression analysis of TERG_08771 showed a fluctuation profile during the co-culture of *T. rubrum* conidia with keratinocytes. This modulation may be directly involved in the cell cycle and possibly in conidia structures.

The genomes of dermatophytes are enriched in genes containing the carbohydrate-binding domain LysM, presenting a differential modulation in gene expression throughout *T. rubrum* growth in response to nutrient sources [52]. Furthermore, TERG_05627 and TERG_01873 were able to bind chitin and human N-linked oligosaccharides from skin glycoproteins. Thus, they may contribute to fungal adhesion and evasion from the innate host defense by shielding chitin structures [53]. Moreover, in *Arthroderma* *benhamiae*, hydrophobin HypA is a cell surface protein involved in fungal evasion from host recognition by neutrophils [41]. In *T. rubrum*, the *hypA* gene is highly expressed during growth in keratin, among other cell wall-related genes, such as the *scw11* (glycosyl hydrolase, glucanase) and the *sur1* (mannosyl phosphorylinositol ceramide synthase) [23,54]. Moreover, the *hypA* gene is regulated by the transcription factor StuA, which also controls morphogenesis, keratinolytic activity, and stress response [23]. Furthermore, the release of extracellular vesicles from *T. interdigitale* modulates keratinocyte and macrophage responses by inducing a proinflammatory response and enhancing the phagocytic and fungicidal activity of macrophages [26].

Conidia are unicellular structures with low metabolic activity that remain quiescent until optimal conditions for germination are achieved [42,51]. The quiescent status of the conidia may be an additional adaptive strategy for fungal survival and establishment of infection within the host. Indeed, it has been shown that dormant *T. rubrum* conidia work as a warehouse of a pre-existing pool of RNAs and proteins involved in conidia dormancy, maintenance, and germination [51,55]. Among the proteins identified were those involved in cell wall assembly and remodeling, proteins related to rodlet layer deposition in the surrounding conidia, signaling transduction pathways that govern nutrient sensing, and regulation of polarized growth. Although dormant conidia are characterized by low metabolic activity in a quiescent state, the aforementioned studies identified many proteins related to energy metabolism and protein synthesis. These data reinforce the complexity of the conidia molecular network, which assures a rapid response of the conidia according to environmental cues. In this sense, conidia germination occurs rapidly under adverse conditions to prevent fungal elimination by host defenses or desquamation, ultimately allowing hyphae formation and penetration.

During penetration into the host tissue, fungi must search for and scavenge nutrients to ensure the establishment of infection, while several proteins are secreted, such as lipases, phosphatases, and keratinolytic proteases [56]. The proteolytic degradation of keratinized structures results in short oligopeptides and free amino acids taken up by fungal cells. In addition, the degradation of hard keratin, such as keratin located in nails and hairs, is only possible after the relaxation of its structure, which is mediated by sulfite action that reduces disulfide bridges. Sulfite is secreted by a sulfite efflux pump (encoded by the *ssu1* gene) whose role in loosening the keratin structure is well known [57]. Moreover, the production of cysteine is cited as a consequence of keratin degradation, which must be regulated within the cells. This is achieved by the enzymatic activity of cysteine dioxygenase type 1, which metabolizes cysteine in sulfite and facilitates keratin degradation [57,58].

A recent report evaluated the primary metabolites obtained from *T. rubrum* and *M. canis* under growth in glucose and keratin sources [59]. This study highlighted the presence of cysteine, alanine, kynurenic acid, and riboflavin during keratin growth, and oxaloacetate, uracil, hydroxyproline, pyridoxine, and glutathione when glucose was used as a nutrient. These compounds crosslink with energy pathways, such as the tricarboxylic acid (TCA) cycle, glycolysis, and the amino acid degradation pathway. This work demonstrated particularities in the metabolome profile of these two genera, which may affect adaptation to specific niches for each genus or species of dermatophytes, and how they cope with environmental stress and nutrient availability. In addition, the time-course transcriptional profile of *T. rubrum* mycelial growth in minimal medium containing glucose or keratin showed the downregulation of genes related to glycolysis, nitrogen catabolism, and the TCA cycle, and the upregulation of glyoxylate genes, such as *acuD* (isocitrate lyase)*,* in keratin growth [60].

Furthermore, this study highlighted that keratin degradation is followed by high levels of ammonium production and ultimately by mechanisms related to glutamine and urea metabolism that are activated for ammonium utilization and extrusion. Moreover, the genes encoding citrate synthase (*citA*) and malate synthase (*acuE*) were upregulated during *T. rubrum* growth in keratin and nail; *citA* was also upregulated during ex vivo human skin infection [25]. However, deletion of the genes *acuD* and *acuE* in *A. benhamiae* did not alter virulence in guinea pigs or reconstituted human epidermal infections [61].

### 2.2. Transcription Factors and Fungal Signaling Pathways as Virulence Traits

During infection, a complex and orchestrated circuit of intracellular signaling is activated to regulate responsive genes involved in the adherence, penetration, and maintenance of dermatophytes in the host environment [24]. The first interaction of dermatophytes with skin and nails occurs under acidic pH conditions. The maintenance of skin pH at an average of 4.7, is related to defense against infections [62,63]. It is promoted by combining molecules, such as acid lipids, amino acids, free fatty acids from glands and epidermal cells, and resident microbiota [24,64]. Although the profound influence of pH on the growth of microorganisms has been extensively discussed, many aspects regarding the molecular signaling response to environmental pH require further investigation. The current knowledge about pH sensing during dermatophyte infection shows that initial contact with skin triggers the de-repression of genes coding for proteases, lipases, adhesins, and acetamidase, among others, which display optimum activity at acidic pH values. Due to protein degradation and metabolism, such as glycine and acetate, a shift from acidic to alkaline pH is achieved. The overexpression of genes that encode proteases and transporters with optimum alkaline activity has been demonstrated [36,65]. Notably, this shift is dependent on the carbon source and is affected by the initial environmental pH.

In this context, the signaling pathways that transduce the extracellular pH and allow a prompt response to these changes are relevant for adaptation, survival, growth, dissemination in different niches, and virulence [64,66]. The transcription factor PacC, a well-known pH response pathway component, plays a role in pathogenesis and immune modulation during fungal infection [66]. In dermatophytes, PacC plays a role in virulence, including protease secretion, keratinolytic activity, and growth in human nails [67]. Disruption of the *pacC* gene in *T. interdigitale* (previously identified as *T. rubrum* H6 and reclassified based on genome sequencing [68]) did not change the extracellular alkalinization during keratin growth, nor did the modulation of carboxypeptidase or acetamidase genes, both involved in keratin metabolism and alkalinization [69]. From this observation, we assumed that the decrease in alkaline protease secretion in the Δ*pacC* strain was not related to the alkalinization of the culture medium.

The PacC signaling pathway comprises seven proteins, PalH, PalI, PalF, PalC, PalA, and PalB, which convey any change in environmental pH to the transcription factor PacC, which commences at neutral to alkaline pH. The external pH is sensed by a membrane complex comprising three proteins, PalH (putative sensor), PalI, and PalF (assistant proteins). Subsequently, in *Aspergillus nidulans*, PalA, PalB, and PalC interact with endosomal sorting complexes required for transport (ESCRT) proteins, which is followed by activation of PalB (signaling protease) and the subsequent two-step proteolytic cleavage of PacC (a 72 kDa full-length protein) yielding an N-terminal 53 kDa protein, PacC^53^, and then the 27 kDa final product, PacC^27^ [70,71]. In this sense, it was supposed that the full-length PacC protein would be inactive under acidic conditions. Nevertheless, assays carried out in *A. nidulans* demonstrated the activity of full-length PacC under acidic pH [72]. In addition, a recent study that profiled the gene modulation governed by Pac3 in the filamentous fungus *Neurospora crassa* demonstrated this transcription factor’s diverse metabolic and adaptive roles and its impact on the regulation of an additional 12 transcription factors [73]. Previous studies in dermatophytes have shown the involvement of the PacC signaling cascade in post-translational modifications (PTM), mainly related to glycosylation [74]. In this respect, glycosylation of the enzyme phosphatase (Pho-2) was identified among the metabolic responses to pH identified in *A. nidulans* [75]. Although the amount of Pho-2 produced was the same irrespective of growth pH (pH 5.4 or pH 7.8), the enzyme activity detected at alkaline pH differs from that at acidic pH, which is due to the lower glycosylation level at acidic pH [76]. Indeed, changes in protein glycosylation affect their stability and affinity to their substrates.

All genes belonging to the PacC signaling pathway were identified in dermatophyte genomes, suggesting the conservation of this cascade among these fungi [64]. As mentioned above, PacC in *T. interdigitale* is involved in protease activity and growth on the host keratinized molecules [67]. Furthermore, another study demonstrated the role of PacC in the regulation of N- and O-linked mannosyltransferases in *T. interdigitale*. This work showed that under different pH values (pH 5.0 or pH 8.0), the modulation of N-mannosyltransferase and O-mannosyltransferase genes are affected differently in a Δ*pacC*-background [74]. Thus, decreased keratinolytic activity may be related to changes in protease glycosylation. In addition, crosstalk with other pathways with PacC signaling has been advocated to assure cellular homeostasis and the levels of Na^+^ and K^+^ [77].

A highly developed secretory system is critical for fungal virulence. This system ensures the delivery of hydrolytic enzymes, transporters, and other proteins into and across the cell membrane, allowing attachment to host tissues and nutrient uptake by the pathogen. The endoplasmic reticulum (ER) is the gateway for protein secretion, which provides proper protein folding, modification, and extracellular protein export. These functions are guaranteed through the action of resident chaperones, foldases, and PTM enzymes [78]. The high demand for protein secretion overwhelms the ER capacity, compromising its function. Thus, to mitigate the ensuing status of ER stress, an unfolded protein response (UPR) pathway is activated [79]. This pathway is composed of two proteins, an ER-transmembrane sensor Ire1/IreA (Ser/Thr kinase) with an endonuclease domain, and a transcription factor, Hac1/HacA. Upon ER stress, the IreA sensor protein is self-activated, which in turn activates the transcription factor HacA through a non-canonical splice in conserved splice sites of a hairpin RNA secondary structure. The activated form of HacA, containing a bZIP domain, is directed from the cytoplasm to the nucleus to prompt the regulation of UPR target genes [80]. In *T. rubrum*, deletion of the *hacA* gene resulted in a strain that was more susceptible to antifungal compounds, such as azoles and cell wall disrupting agents, and that presented a reduction in growth on human nail fragments and keratinocytes. In addition, the N-mannan and alfa-mannan encoding genes were upregulated in this strain as compared with the wild-type. Indeed, the search for putative HacA target genes showed that HacA may regulate approximately 25% of the *T. rubrum* genome. Among the UPR target genes are the genes that encode mannosyltransferase enzymes, heat shock proteins (Hsps), fatty acid biosynthetic enzymes, cell wall enzymes, and proteases [22].

As mentioned above, PacC and HacA are involved in mannosyltransferase regulation, and both transcription factors are also related to Hsp regulation [22,24]. Hsps are conserved chaperones with multiple roles in the cell, such as aiding the folding and transport of proteins, protection under stressful conditions, and fungal pathogenicity. In yeasts, their regulation is dependent on two regulatory sequences, the stress response elements (STRE) and the heat shock elements (HSE), which are binding sites for the transcription factors Msn24p and Hsf1p, respectively [81,82,83]. During interaction with host molecules, some Hsp-encoding genes were upregulated in *T. rubrum,* such as *hsp30*, *hsp104*, and *hsp75-like,* as shown after co-culture with keratinocytes [84], and the overexpression of *hsp60*, *hsp70*, and *hsp78* genes was promoted through interaction with human nail fragments [85,86]. Moreover, three genes that encode putative Hsp70 proteins were also identified in *A. benhamiae* after exposure to keratin [38].

Inhibition of Hsp90 by the synthetic compound 17-AGG (17-allylamino-17-demethoxygeldanamycin) caused a severe compromise in nail infection by *T. rubrum* and impaired the keratinolytic activity at 37 °C [84,85]. There is a relationship between PacC and Hsf1 in the production of Hsp transcripts. The growth of *T. interdigitale* in keratin increased the transcript levels of both *hsf1* and *pacC* genes in the wild-type strain, whereas the *hsf1* gene was downregulated in the Δ*pacC* strain. The transcription levels of *hsp75-like* and *hsp90* are also regulated by HacA [22]. Conceivably, the production of Hsps is controlled by PacC, Hsf1, and HacA.

Furthermore, the APSES family of transcription regulators (Asm1p, Phd1p, Sok2p, Efg1p, and StuA) regulates different cellular processes, including heat shock tolerance in dermatophytes. Recent studies investigating the StuA functionality in *T. rubrum* demonstrated its role in virulence, hydrophobicity, stress tolerance, and a deep involvement in physiology by regulating central carbon metabolism, glycerol catabolism, reactive oxygen species metabolism, and cell wall construction [23,87]. In silico analysis predicted that StuA can control the expression of 17% of the total genome of this dermatophyte and is involved in various biological processes, such as oxidation-reduction, phosphorylation, proteolysis, transcription/translation regulation, and carbohydrate metabolism [23]. Additionally, StuA is involved in keratin degradation and reproduction in *A. benhamiae* [88].

**Figure 1:**
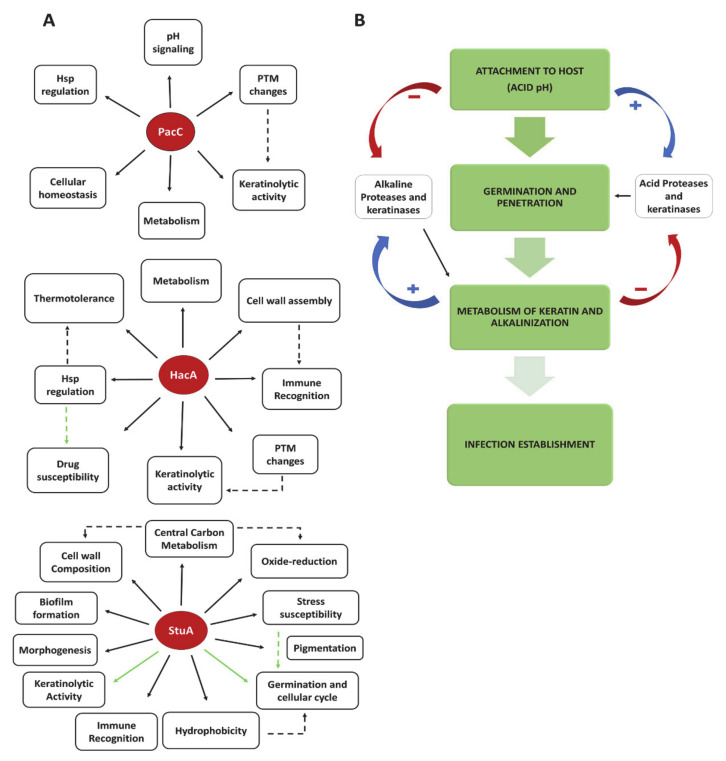
Diagram displaying proteins and enzymes involved in the infectious process: (**A**) The functionality of three transcription factors assessed in dermatophytes and the convergence of their roles during fungus-host interaction and infection outcome. Solid black arrows represent the current knowledge about the regulated processes. Dashed black arrows indicate a correlation between the regulated functions. Solid green arrows depict the possible interwoven paths that control some downstream effects. Dashed green arrows indicate a hypothesized correlation of processes through cross-related paths yet to be elucidated. PTM is related to post-translational modifications; (**B**) stages in the dermatophyte infection process. Positive and negative signals represent induction or repression, respectively.

Indeed, understanding the functionality of fungal transcription factors and their physiological and mechanistic roles has garnered special attention [89]. Transcription factors comprise the last link between signal perception and activation of targeted genes. In this respect, the repertoire of transcription factors coordinates cell behavior and governs life and adaptation. They regulate pathways involved in adhesion, conidiation, nutrient acquisition, adaptation to environmental stress, and the interplay between fungi and the host. Much of the knowledge regarding transcription factor functionality has been provided through genetic studies. In this sense, we have unveiled pieces of this puzzle for some transcription factors in dermatophytes (Figure 1), although the complete picture of the role of transcription factors during in vivo and in vitro infection is enigmatic and remains to be fully addressed. Different infection models have been valuable tools for managing the pathophysiological properties of transcription factors and many genes during the host-pathogen interaction process.

## 3. Treatment, Clinical Implications, and Perspectives

### 3.1. Therapeutic Options and Resistance Mechanisms

**Figure 2:**
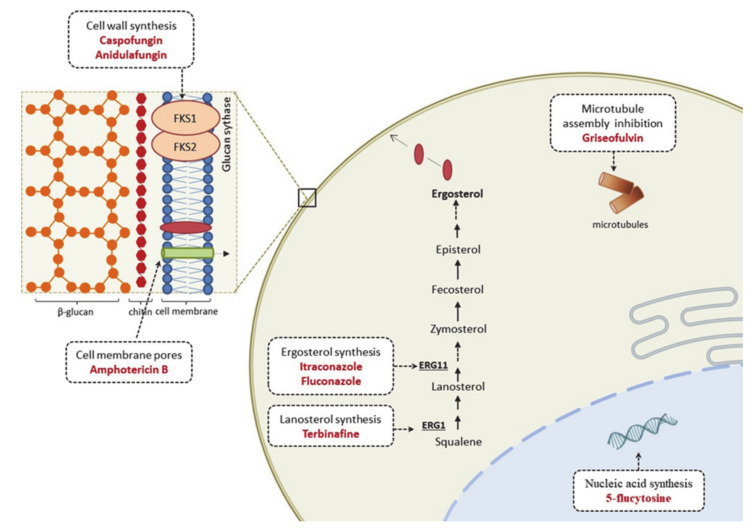
Mode of action of antifungal drugs.

Antifungals are limited to a few structural classes of drugs, including allylamines, polyenes, azoles, echinocandins, and other agents, such as griseofulvin and 5-flucytosine (Figure 2). However, echinocandins and 5-flucytosine are used only for invasive fungal infections and not for dermatophytosis. In general, the drugs used for dermatophytosis treatment target the ergosterol biosynthetic pathway, specifically in enzymes related to the biosynthesis of this major fungal membrane sterol. Allylamine terbinafine, a squalene epoxidase inhibitor with fungicidal activity, is highly effective against dermatophytes [90,91]. It is the first-line treatment for dermatophyte infections, such as tinea unguium and tinea capitis in children infected with *Trichophyton* spp. However, griseofulvin, a fungistatic drug that inhibits microtubule assembly and ultimately affects mitosis, may be a better choice for tinea capitis caused solely by *Microsporum* spp. [92,93]. Azoles, such as itraconazole and fluconazole, may also be used for dermatophytosis treatment. Both are triazole antifungal agents with fungistatic activity, inhibiting the cytochrome P450 enzyme lanosterol 14α-demethylase [33].

**Figure 3:**
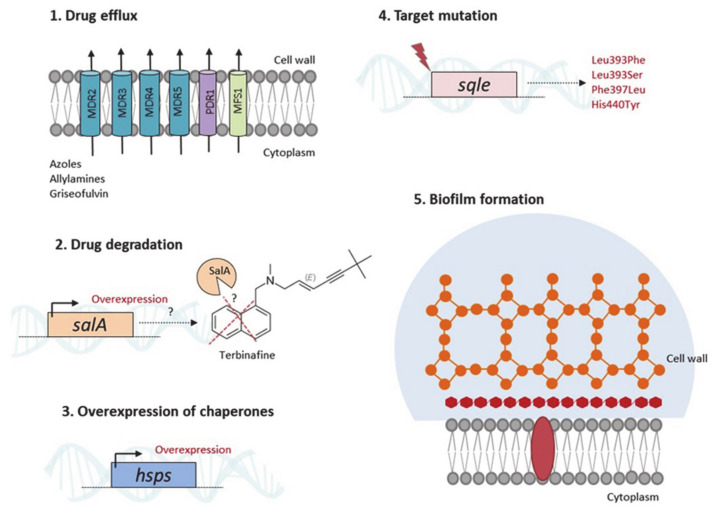
Antifungal resistance mechanisms described in dermatophytes.

Although treatment is available, there are increasing reports of antifungal resistance and tolerance in dermatophytes [33,94]. *Trichophyton rubrum* is the most predominant dermatophyte species and is the most recurrently described in resistance to standard treatments, followed by *T. interdigitale* [19]. Long-term and discontinuing treatments may lead to recalcitrant infections, thus favoring the acquisition of resistance. Genetic and biochemical mechanisms of antifungal resistance have been reported in dermatophytes, including point mutations, alteration in drug target sites, and increased efflux-mediated activity to the currently available drugs [33,95,96]. The reduced antifungal potency of terbinafine may be due to single-point mutations in the squalene epoxidase (*sqle*) target gene. In *T. rubrum*, Leu393Phe, Leu393Ser, Phe397Leu, and His440Tyr amino acid substitutions in the Sqle have been associated with terbinafine resistance [97]. The authors also showed that while terbinafine minimal inhibitory concentrations (MIC) were similar for the isolates harboring Leu393Phe, those carrying Phe397Leu presented significantly different MIC values. In a recent analysis of terbinafine resistance in *T. mentagrophytes*, 91% of the isolates presented the Phe397Leu amino acid substitution in the Sqle, and the isolated strains displayed high terbinafine MIC values [94]. In addition, the highest MIC values were observed for isolates carrying Leu393Phe substitutions. This study also showed that 42% of terbinafine-sensitive isolates were resistant to itraconazole and voriconazole. Higher MIC values for these two triazoles were observed in isolates carrying the Ala448Thr substitution [94]. It has been proposed that prolonged drug exposure during treatment could favor the emergence of resistant isolates [97,98,99]. Interestingly, in *Trichophyton indotineae*, an anthropophilic species belonging to the *T. mentagrophytes*/*T. interdigitale* species complex, epidemic in North India and highly terbinafine resistant, was also detected a missense mutation (Phe397Leu) in the *sqle* gene [100,101]. Another mechanism that accounts for terbinafine resistance is an increase in drug degradation by salicylate-1-monoxigenase (SalA), as previously reported for *T. rubrum*, in which the *salA* gene was upregulated in response to terbinafine, and additional copies of the *salA* gene conferred terbinafine resistance when introduced into a susceptible strain by transformation [34] (Figure 3).

Antifungal cross-resistance has also been reported in dermatophytes, revealing concomitantly reduced sensitivity to different classes of drugs, including allylamines and azoles, the second choice for clinical treatment. A recent study revealed that terbinafine-resistant *T. interdigitale* isolates are cross-resistant to fluconazole, sertaconazole, itraconazole, voriconazole, and griseofulvin [99]. Cross-resistance was observed in a terbinafine-resistant *T. rubrum* isolate, which was also resistant to azoles associated with the overexpression of the multidrug efflux transporter TruMDR3 [102]. Multidrug resistance and response mediated by the efflux activity of transporters have been reported in dermatophytes, including the ATP-binding cassette (ABC) family of multidrug resistance transporters MDR2, MDR4, and MDR5, and the pleiotropic drug resistance (PDR1). These reports also showed that several drugs, including terbinafine, griseofulvin, and azoles, induced the expression of transporter encoding genes [96,102,103,104,105]. Furthermore, the major facilitator superfamily (MFS) TruMFS1 transporter was identified, conferring resistance to azoles in dermatophytes [102]. These findings show that although the efflux activity of these proteins presents considerable overlap among different antifungal drugs, specificity is also observed in resistance mediated by these transporters. In addition, these studies indicated that even without directly participating in drug resistance, increased expression of transporter-encoding genes might be an adaptive mechanism to respond quickly and cope with the toxic effects of antifungal drugs, compensating for the absence of other transporters [102]. Efflux activity may also be species specific, making it more challenging to choose the right drug against dermatophytes.

Dermatophytes also produce biofilms in the host tissue, which may account for clinical resistance, treatment failure, and the occurrence of recalcitrant infections. The current understanding of dermatophyte biofilm-mediated resistance is scarce. Recently, it was shown that in *T. mentagrophytes* and *M. canis*, terbinafine, griseofulvin, and itraconazole, MIC values were higher for biofilms produced in vitro than for planktonic cells. In addition, biofilms produced ex vivo using cat hairs have been shown to be more tolerant to these antifungal drugs than biofilms produced in vitro [106]. This study highlights that nutritional source and availability also influence drug tolerance in biofilms and resistance in dermatophytes, as previously demonstrated [107]. Terbinafine has been observed to have a high inhibitory effect against *T. rubrum* during in vitro biofilm formation. However, for mature biofilms, amphotericin B exerted a more significant inhibitory effect than terbinafine [108]. These results indicate a variable response to conventional therapy during biofilm formation (Figure 3).

The routine and uncontrolled use of antifungal drugs also parallels the development of resistance. To overcome this, the first option is to expand clinical treatment against dermatophytes with promising candidates already available, broadening the possibilities of therapy. The echinocandin drug, anidulafungin, was potent against dermatophytes in vitro. Echinocandins are inhibitors of fungal cell wall synthesis, broadening the options for effective antifungal agents against dermatophytes [90]. However, currently, echinocandins exist only in an intravenous form which is not recommended for treating dermatophytosis. But, the number of azoles used for clinical has increased. Newer broad-spectrum agents of the triazole series include posaconazole, isavuconazole, voriconazole, efinaconazole, luliconazole, and lanaconazole. They possess potent in vitro antifungal activity against dermatophytes, many of which are more efficient than the commonly used antifungal agents. However, further studies are warranted to determine the relevance of these in vitro findings in clinical efficacy [91,109,110]. The treatment of dermatophyte infections is challenging, and alternatives must be explored to overcome tolerance and resistance while new antifungal drugs are investigated.

### 3.2. From Knowing the Enemy to Identifying Novel Antifungal Targets

Historically, the identification of antifungal-active substances relies on the screening of synthetic molecules or natural compounds and evaluating their ability to inhibit in vitro growth of selected fungal pathogens. However, these substances may also confer toxicity to the host because of the closeness between fungal and human cells in terms of structure and biochemical processes, thus, restricting drug development approaches [111]. Targeting genes that are essential for fungal viability but are not found in mammals, or genes that provide characteristic pathogenic traits, has prompted researchers to seek new antifungal agents and therapeutic strategies.

Advances in molecular methodologies and genetic manipulations in dermatophytes have enabled testing hypotheses regarding genes’ essentiality, functionality, and regulation in several aspects of dermatophytes biology. The transcription factor StuA is an example of a gene unique to fungi with a singular function in dermatophytes. Deletion of this transcription factor, which is involved in various cellular processes, impacts *T. rubrum* virulence and physiology [23,87] and sexual reproduction in *A. benhamiae* [88]. Deletion also resulted in impaired growth and reduced aerial hyphae production during cultivation in solid media [23]. These results revealed the role of StuA in both anthropophilic and zoophilic dermatophyte species, highlighting its potential as a target for the development of antifungal drugs. Furthermore, disruption of the gene coding for the transcription factor Dnr1 in *M. canis*, which is homologous to the nitrogen regulatory genes *areA* from *A. nidulans* and *nit-2* from *N. crassa,* impaired growth in keratin [112]. The deletion of a gene homologous to the *areA/nit2* in *T. mentagrophytes* (*tnr*) delayed the infectious ability in Guinea pigs. [113]. In ascomycetes, this transcription factor is a crucial regulator of nitrogen metabolite repression and is required for full virulence [114], indicating another relevant molecular target for drug development.

High-throughput RNA-sequencing analysis has boosted the understanding of gene function and regulation. Transcriptomic analysis depicts how genes are globally regulated under diverse conditions, surpassing the punctual evaluation of the effects of a single gene on the fungal phenotype and its interconnection with a few selected genes. More than gene expression, the advent of high-throughput sequencing has uncovered diverse mature mRNAs derived from a single gene through alternative splicing (AS), a post-transcriptional regulation mechanism. Exploring AS events in the *T. rubrum* transcriptome under exposure to undecanoic acid has revealed intron retention events in several genes, including the *pakA* gene, a potential virulence factor in fungi [32]. The analysis resulted in a feasible and novel molecular mechanism for activating Ste20/PakA kinase based on an alternative pre-mRNA splicing process, resulting in a translation event of Ste20/PakA kinase free of its autoinhibitory CRIB (Cdc42/Rac-interactive binding) domain. These findings highlight Ste20/PakA as a potential target for new drugs against dermatophytes [115].

Intron retention events in Hsp-encoding genes were likewise identified from the RNA-seq of *T. rubrum* exposed to undecanoic acid. The results suggest that Hsp70 family members are relevant candidates for post-transcriptional regulation by AS during development and response to extracellular stimuli in the dermatophyte, possibly providing adaptive advantages to the fungus. These findings also indicate that Hsp network proteins are potential targets for drug discovery [84]. Indeed, *T. rubrum hsp90*, *hsp88-like, hsp20, hsp60, hsp70,* and *cdc37* co-chaperone genes were upregulated in response to terbinafine exposure, and chemical inhibition of Hsp90 by 17-AAG decreased fungal growth in nails and displayed a synergistic effect with itraconazole and micafungin [85,86]. Although promising insights have been provided by these studies that led to the identification of putative cellular targets for the development and screening of new antifungal drugs, the feasibility of the potential targets requires further investigation.

## 4. Conclusions

Global warming, increasing travel and migration, socioeconomic problems, and contact with pets may explain the increase in the incidence of dermatophytosis worldwide in recent decades. Cutaneous mycoses are among the most prevalent fungal diseases in humans. This review discussed the latest findings on dermatophyte physiology, virulence traits, and antifungal resistance. Combining large-scale transcriptomic analysis and functional genetics has enabled the development of regulatory molecular models to assess the dynamic behavior of the pathogenesis of dermatophytes and antifungal resistance. During infection, dermatophytes sense the host tissue, triggering signaling pathways that culminate in gene expression changes and metabolic adaptation to enable fungal invasion, survival, and dissemination. Moreover, exposure to antifungal drugs induces fungal biochemical and genetic mechanisms to overcome their toxic effects. In addition, the misuse and abuse of antifungal and treatment discontinuation are factors that contribute to the increasing resistance of pathogens. Although several advances have been achieved in the field, we are still far from solving the problem, which requires the urgent need for more studies and the awareness to stop neglecting fungal diseases. Taken together, these findings provide further insights into new approaches to prevent, control, and treat dermatophytosis.

## References

[1] De Hoog S., Monod M., Dawson T., Boekhout T., Mayser P., Gräser Y. (2017). Skin Fungi from Colonization to Infection. Microbiol. Spectr..

[2] Mushtaq S., Faizi N., Amin S.S., Adil M., Mohtashim M. (2020). Impact on quality of life in patients with dermatophytosis. Australas. J. Dermatol..

[3] Narang T., Bhattacharjee R., Singh S., Jha K., Kavita K., Mahajan R., Dogra S. (2019). Quality of life and psychological morbidity in patients with superficial cutaneous dermatophytosis. Mycoses.

[4] Kovitwanichkanont T., Chong A.H. (2019). Superficial fungal infections. Aust. J. Gen. Pract..

[5] Dogra S., Narang T. (2017). Emerging atypical and unusual presentations of dermatophytosis in India. Clin. Dermatol. Rev..

[6] Atzori L., Pau M., Aste N., Aste N. (2012). Dermatophyte infections mimicking other skin diseases: A 154-person case survey of tinea atypica in the district of Cagliari (Italy). Int. J. Dermatol..

[7] Boral H., Durdu M., Ilkit M. (2018). Infection and Drug Resistance Majocchi’s granuloma: Current perspectives. IDR.

[8] Rouzaud C., Hay R., Chosidow O., Dupin N., Puel A., Lortholary O., Lanternier F. (2015). Severe Dermatophytosis and Acquired or Innate Immunodeficiency: A Review. J. Fungi.

[9] Agarwal A., Hassanandani T., Das A., Panda M., Chakravorty S. (2021). “Mask tinea”: Tinea faciei possibly potentiated by prolonged mask usage during the COVID-19 pandemic. Clin. Exp. Dermatol..

[10] Gnat S., Nowakiewicz A., Łagowski D., Zięba P. (2019). Host- and pathogen-dependent susceptibility and predisposition to dermatophytosis. J. Med. Microbiol..

[11] Gnat S., Łagowski D., Nowakiewicz A. (2021). Genetic Predisposition and Its Heredity in the Context of Increased Prevalence of Dermatophytoses. Mycopathologia.

[12] Lanternier F., Pathan S., Vincent Q.B., Liu L., Cypowyj S., Prando C., Migaud M., Taibi L., Ammar-Khodja A., Stambouli O.B. (2013). Deep dermatophytosis and inherited CARD9 deficiency. N. Engl. J. Med..

[13] Begum J., Mir N.A., Lingaraju M.C., Buyamayum B., Dev K. (2020). Recent advances in the diagnosis of dermatophytosis. J. Basic Microbiol..

[14] De Hoog G.S., Dukik K., Monod M., Packeu A., Stubbe D., Hendrickx M., Kupsch C., Stielow J.B., Freeke J., Göker M. (2017). Toward a Novel Multilocus Phylogenetic Taxonomy for the Dermatophytes. Mycopathologia.

[15] Verma S.B., Panda S., Nenoff P., Singal A., Rudramurthy S.M., Uhrlass S., Das A., Bisherwal K., Shaw D., Vasani R. (2021). The unprecedented epidemic-like scenario of dermatophytosis in India: I. Epidemiology, risk factors and clinical features. Indian J. Dermatol. Venereol. Leprol..

[16] Gupta A.K., Stec N., Summerbell R.C., Shear N.H., Piguet V., Tosti A., Piraccini B.M. (2020). Onychomycosis: A review. J. Eur. Acad. Dermatol. Venereol..

[17] Rodríguez-Cerdeira C., Martínez-Herrera E., Szepietowski J.C., Pinto-Almazán R., Frías-De-León M.G., Espinosa-Hernández V.M., Chávez-Gutiérrez E., García-Salazar E., Vega-Sánchez D.C., Arenas R. (2021). A systematic review of worldwide data on tinea capitis: Analysis of the last 20 years. J. Eur. Acad. Dermatol. Venereol..

[18] Sacheli R., Harag S., Dehavay F., Evrard S., Rousseaux D., Adjetey A., Seidel L., Laffineur K., Lagrou K., Hayette M.-P. (2020). Belgian National Survey on Tinea Capitis: Epidemiological Considerations and Highlight of Terbinafine-Resistant *T. mentagrophytes* with a Mutation on SQLE Gene. J. Fungi.

[19] Bontems O., Fratti M., Salamin K., Guenova E., Monod M. (2020). Epidemiology of Dermatophytoses in Switzerland According to a Survey of Dermatophytes Isolated in Lausanne between 2001 and 2018. J. Fungi.

[20] Wang R., Huang C., Zhang Y., Li R. (2021). Invasive dermatophyte infection: A systematic review. Mycoses.

[21] Mercer D.K., Stewart C.S. (2019). Keratin hydrolysis by dermatophytes. Med. Mycol..

[22] Bitencourt T.A., Lang E.A.S., Sanches P.R., Peres N.T.A., Oliveira V.M., Fachin A.L., Rossi A., Martinez-Rossi N.M. (2020). HacA Governs Virulence Traits and Adaptive Stress Responses in *Trichophyton rubrum*. Front. Microbiol..

[23] Lang E.A.S., Bitencourt T.A., Peres N.T.A., Lopes L., Silva L.G., Cazzaniga R.A., Rossi A., Martinez-Rossi N.M. (2020). The *stuA* gene controls development, adaptation, stress tolerance, and virulence of the dermatophyte *Trichophyton rubrum*. Microbiol. Res..

[24] Martinez-Rossi N.M., Peres N.T.A., Rossi A. (2017). Pathogenesis of Dermatophytosis: Sensing the Host Tissue. Mycopathologia.

[25] Peres N.T.A., Silva L.G., Santos R.S., Jacob T.R., Persinoti G.F., Rocha L.B., Falcão J.P., Rossi A., Martinez-Rossi N.M. (2016). In vitro and ex vivo infection models help assess the molecular aspects of the interaction of *Trichophyton rubrum* with the host milieu. Med. Mycol..

[26] Bitencourt T.A., Rezende C.P., Quaresemin N.R., Moreno P., Hatanaka O., Rossi A., Martinez-Rossi N.M., Almeida F. (2018). Extracellular Vesicles from the Dermatophyte *Trichophyton interdigitale* Modulate Macrophage and Keratinocyte Functions. Front. Immunol..

[27] Burstein V.L., Beccacece I., Guasconi L., Mena C.J., Cervi L., Chiapello L.S. (2020). Skin Immunity to Dermatophytes: From Experimental Infection Models to Human Disease. Front. Immunol..

[28] Gupta A.K., Foley K.A., Versteeg S.G. (2017). New Antifungal Agents and New Formulations Against Dermatophytes. Mycopathologia.

[29] Martinez-Rossi N.M., Bitencourt T.A., Peres N.T.A., Lang E.A.S., Gomes E.V., Quaresemin N.R., Martins M.P., Lopes L., Rossi A. (2018). Dermatophyte Resistance to Antifungal Drugs: Mechanisms and Prospectus. Front. Microbiol..

[30] Rossi A., Martins M.P., Bitencourt T.A., Peres N.T.A., Rocha C.H.L., Rocha F.M.G., Neves-da-Rocha J., Lopes M.E.R., Sanches P.R., Bortolossi J.C. (2021). Reassessing the Use of Undecanoic Acid as a Therapeutic Strategy for Treating Fungal Infections. Mycopathologia.

[31] Persinoti G.F., de Peres N.T.A., Jacob T.R., Rossi A., Vêncio R.Z., Martinez-Rossi N.M. (2014). RNA-sequencing analysis of *Trichophyton rubrum* transcriptome in response to sublethal doses of acriflavine. BMC Genom..

[32] Mendes N.S., Bitencourt T.A., Sanches P.R., Silva-Rocha R., Martinez-Rossi N.M., Rossi A. (2018). Transcriptome-wide survey of gene expression changes and alternative splicing in *Trichophyton rubrum* in response to undecanoic acid. Sci. Rep..

[33] Khurana A., Sardana K., Chowdhary A. (2019). Antifungal resistance in dermatophytes: Recent trends and therapeutic implications. Fungal Genet. Biol..

[34] Santos H.L., Lang E.A.S., Segato F., Rossi A., Martinez-Rossi N.M. (2018). Terbinafine resistance conferred by multiple copies of the salicylate 1-monooxygenase gene in *Trichophyton rubrum*. Med. Mycol..

[35] Paião F.G., Segato F., Cursino-Santos J.R., Peres N.T.A., Martinez-Rossi N.M. (2007). Analysis of *Trichophyton rubrum* gene expression in response to cytotoxic drugs. FEMS Microbiol. Lett..

[36] Maranhão F.C.A., Paião F.G., Martinez-Rossi N.M. (2007). Isolation of transcripts over-expressed in human pathogen *Trichophyton rubrum* during growth in keratin. Microb. Pathog..

[37] Maranhão F.C.A., Silveira H.C.S., Rossi A., Martinez-Rossi N.M. (2011). Isolation of transcripts overexpressed in the human pathogen *Trichophyton rubrum* grown in lipid as carbon source. Can. J. Microbiol..

[38] Staib P., Zaugg C., Mignon B., Weber J., Grumbt M., Pradervand S., Harshman K., Monod M. (2010). Differential gene expression in the pathogenic dermatophyte *Arthroderma benhamiae* in vitro versus during infection. Microbiology.

[39] De Faria L.V., do Carmo P.H.F., da Costa M.C., Peres N.T.A., Rodrigues Chagas I.A., Furst C., Ferreira G.F., Costa A.O., Santos D.A. (2020). *Acanthamoeba castellanii* as an alternative interaction model for the dermatophyte *Trichophyton rubrum*. Mycoses.

[40] Campos M.R.M., Russo M., Gomes E., Almeida S.R. (2006). Stimulation, inhibition and death of macrophages infected with *Trichophyton rubrum*. Microbes Infect..

[41] Heddergott C., Bruns S., Nietzsche S., Leonhardt I., Kurzai O., Kniemeyer O., Brakhage A.A. (2012). The *Arthroderma benhamiae* hydrophobin HypA mediates hydrophobicity and influences recognition by human immune effector cells. Eukaryot. Cell.

[42] Faway É., Lambert de Rouvroit C., Poumay Y. (2018). In vitro models of dermatophyte infection to investigate epidermal barrier alterations. Exp. Dermatol..

[43] Ishii M., Matsumoto Y., Yamada T., Abe S., Sekimizu K. (2017). An invertebrate infection model for evaluating anti-fungal agents against dermatophytosis. Sci. Rep..

[44] Baltazar L., de M., Santos P.C., de Paula T.P., Rachid M.A., Cisalpino P.S., Souza D.G., Santos D.A. (2014). IFN-γ impairs *Trichophyton rubrum* proliferation in a murine model of dermatophytosis through the production of IL-1β and reactive oxygen species. Med. Mycol..

[45] Johns L.E., Goldman G.H., Ries L.N.A., Brown N.A. (2021). Nutrient sensing and acquisition in fungi: Mechanisms promoting pathogenesis in plant and human hosts. Fungal Biol. Rev..

[46] Kaufman G., Horwitz B.A., Duek L., Ullman Y., Berdicevsky I. (2007). Infection stages of the dermatophyte pathogen *Trichophyton*: Microscopic characterization and proteolytic enzymes. Med. Mycol..

[47] Tainwala R., Sharma Y. (2011). Pathogenesis of dermatophytoses. Indian J. Dermatol..

[48] Baldo A., Mathy A., Tabart J., Camponova P., Vermout S., Massart L., Maréchal F., Galleni M., Mignon B. (2010). Secreted subtilisin Sub3 from *Microsporum canis* is required for adherence to but not for invasion of the epidermis. Br. J. Dermatol..

[49] Verstrepen K.J., Jansen A., Lewitter F., Fink G.R. (2005). Intragenic tandem repeats generate functional variability. Nat. Genet..

[50] De Abreu M.H., Bitencourt T.A., Franco M.E., Moreli I.S., Cantelli B.A.M., Komoto T.T., Marins M., Fachin A.L. (2020). Expression of genes containing tandem repeat patterns involved in the fungal-host interaction and in the response to antifungals in *Trichophyton rubrum*. Mycoses.

[51] Bitencourt T.A., Macedo C., Franco M.E., Assis A.F., Komoto T.T., Stehling E.G., Beleboni R.O., Malavazi I., Marins M., Fachin A.L. (2016). Transcription profile of *Trichophyton rubrum* conidia grown on keratin reveals the induction of an adhesin-like protein gene with a tandem repeat pattern. BMC Genom..

[52] Lopes L., Bitencourt T.A., Lang E.A.S., Sanches P.R., Peres N.T.A., Rossi A., Martinez-Rossi N.M. (2020). Genes coding for LysM domains in the dermatophyte *Trichophyton rubrum*: A transcription analysis. Med. Mycol..

[53] Kar B., Patel P., Free S.J. (2019). *Trichophyton rubrum* LysM proteins bind to fungal cell wall chitin and to the N-linked oligosaccharides present on human skin glycoproteins. PLoS ONE.

[54] Martins M.P., Silva L.G., Rossi A., Sanches P.R., Souza L.D.R., Martinez-Rossi N.M. (2019). Global Analysis of Cell Wall Genes Revealed Putative Virulence Factors in the Dermatophyte *Trichophyton rubrum*. Front. Microbiol..

[55] Leng W., Liu T., Li R., Yang J., Wei C., Zhang W., Jin Q. (2008). Proteomic profile of dormant *Trichophyton rubrum* conidia. BMC Genom..

[56] De Peres N.T.A., Maranhão F.C.A., Rossi A., Martinez-Rossi N.M. (2010). Dermatophytes: Host-pathogen interaction and antifungal resistance. An. Bras. Dermatol..

[57] Grumbt M., Monod M., Yamada T., Hertweck C., Kunert J., Staib P. (2013). Keratin degradation by dermatophytes relies on cysteine dioxygenase and a sulfite efflux pump. J. Invest. Dermatol..

[58] Kasperova A., Kunert J., Raska M. (2013). The possible role of dermatophyte cysteine dioxygenase in keratin degradation. Med. Mycol..

[59] Ciesielska A., Kawa A., Kanarek K., Soboń A., Szewczyk R. (2021). Metabolomic analysis of *Trichophyton rubrum* and *Microsporum canis* during keratin degradation. Sci. Rep..

[60] Martins M.P., Rossi A., Sanches P.R., Bortolossi J.C., Martinez-Rossi N.M. (2020). Comprehensive analysis of the dermatophyte *Trichophyton rubrum* transcriptional profile reveals dynamic metabolic modulation. Biochem. J..

[61] Grumbt M., Defaweux V., Mignon B., Monod M., Burmester A., Wöstemeyer J., Staib P. (2011). Targeted gene deletion and in vivo analysis of putative virulence gene function in the pathogenic dermatophyte *Arthroderma benhamiae*. Eukaryot. Cell.

[62] Lambers H., Piessens S., Bloem A., Pronk H., Finkel P. (2006). Natural skin surface pH is on average below 5, which is beneficial for its resident flora. Int. J. Cosmet. Sci..

[63] Wertz P.W., Szalay S. (2020). Innate Antimicrobial Defense of Skin and Oral Mucosa. Antibiotics.

[64] Martinez-Rossi N.M., Persinoti G.F., Peres N.T.A., Rossi A. (2012). Role of pH in the pathogenesis of dermatophytoses. Mycoses.

[65] Peres N.T.A., Sanches P.R., Falco J.P., Silveira H.C.S., Paião F.G., Maranhão F.C.A., Gras D.E., Segato F., Cazzaniga R.A., Mazucato M. (2010). Transcriptional profiling reveals the expression of novel genes in response to various stimuli in the human dermatophyte *Trichophyton rubrum*. BMC Microbiol..

[66] Cornet M., Gaillardin C. (2014). pH signaling in human fungal pathogens: A new target for antifungal strategies. Eukaryot. Cell.

[67] Ferreira-Nozawa M.S., Silveira H.C.S., Ono C.J., Fachin A.L., Rossi A., Martinez-Rossi N.M. (2006). The pH signaling transcription factor PacC mediates the growth of *Trichophyton rubrum* on human nail in vitro. Med. Mycol..

[68] Persinoti G.F., Martinez D.A., Li W., Döğen A., Billmyre R.B., Averette A., Goldberg J.M., Shea T., Young S., Zeng Q. (2018). Whole-Genome Analysis Illustrates Global Clonal Population Structure of the Ubiquitous Dermatophyte Pathogen *Trichophyton rubrum*. Genetics.

[69] Silveira H.C.S., Gras D.E., Cazzaniga R.A., Sanches P.R., Rossi A., Martinez-Rossi N.M. (2010). Transcriptional profiling reveals genes in the human pathogen *Trichophyton rubrum* that are expressed in response to pH signaling. Microb. Pathog..

[70] Díez E., Alvaro J., Espeso E.A., Rainbow L., Suárez T., Tilburn J., Arst H.N., Peñalva M.A. (2002). Activation of the *Aspergillus* PacC zinc finger transcription factor requires two proteolytic steps. EMBO J..

[71] Peñas M.M., Hervás-Aguilar A., Múnera-Huertas T., Reoyo E., Peñalva M.A., Arst H.N., Tilburn J. (2007). Further characterization of the signaling proteolysis step in the *Aspergillus nidulans* pH signal transduction pathway. Eukaryot. Cell.

[72] Rossi A., Cruz A.H.S., Santos R.S., Silva P.M., Silva E.M., Mendes N.S., Martinez-Rossi N.M. (2013). Ambient pH sensing in filamentous fungi: Pitfalls in elucidating regulatory hierarchical signaling networks. IUBMB Life.

[73] Martins M.P., Martinez-Rossi N.M., Sanches P.R., Rossi A. (2020). The PAC-3 transcription factor critically regulates phenotype-associated genes in *Neurospora crassa*. Genet. Mol. Biol..

[74] Mendes N.S., Trevisan G.L., Silva Cruz A.H., Santos R.S., Peres N.T.A., Martinez-Rossi N.M., Rossi A. (2012). Transcription of *N*- and *O*-linked mannosyltransferase genes is modulated by the *pacC* gene in the human dermatophyte *Trichophyton rubrum*. FEBS Open Bio.

[75] Nozawa S.R., Ferreira-Nozawa M.S., Martinez-Rossi N.M., Rossi A. (2003). The pH-induced glycosylation of secreted phosphatases is mediated in *Aspergillus nidulans* by the regulatory gene *pacC*-dependent pathway. Fungal Genet. Biol..

[76] Nozawa S.R., Thede G., Crott L.S.P., Barbosa J.E., Rossi A. (2002). The synthesis of Phosphate-repressible alkaline phosphatase do not appear to be regulated by ambient pH in the filamentous mould *Neurospora crassa*. Braz. J. Microbiol..

[77] Da Silva L.G., Martins M.P., Sanches P.R., de Peres N.T.A., Martinez-Rossi N.M., Rossi A. (2020). Saline stress affects the pH-dependent regulation of the transcription factor PacC in the dermatophyte *Trichophyton interdigitale*. Braz. J. Microbiol..

[78] Römisch K. (2004). A cure for traffic jams: Small molecule chaperones in the endoplasmic reticulum. Traffic.

[79] Moore K.A., Hollien J. (2012). The unfolded protein response in secretory cell function. Annu. Rev. Genet..

[80] Saloheimo M., Valkonen M., Penttilä M. (2003). Activation mechanisms of the HAC1-mediated unfolded protein response in filamentous fungi. Mol. Microbiol..

[81] Wiederrecht G., Seto D., Parker C.S. (1988). Isolation of the gene encoding the *S. cerevisiae* heat shock transcription factor. Cell.

[82] Martínez-Pastor M.T., Marchler G., Schüller C., Marchler-Bauer A., Ruis H., Estruch F. (1996). The *Saccharomyces cerevisiae* zinc finger proteins Msn2p and Msn4p are required for transcriptional induction through the stress response element (STRE). EMBO J..

[83] Boy-Marcotte E., Lagniel G., Perrot M., Bussereau F., Boudsocq A., Jacquet M., Labarre J. (1999). The heat shock response in yeast: Differential regulations and contributions of the Msn2p/Msn4p and Hsf1p regulons. Mol. Microbiol..

[84] Neves-da-Rocha J., Bitencourt T.A., de Oliveira V.M., Sanches P.R., Rossi A., Martinez-Rossi N.M. (2019). Alternative Splicing in Heat Shock Protein Transcripts as a Mechanism of Cell Adaptation in *Trichophyton rubrum*. Cells.

[85] Jacob T.R., Peres N.T.A., Martins M.P., Lang E.A.S., Sanches P.R., Rossi A., Martinez-Rossi N.M. (2015). Heat Shock Protein 90 (Hsp90) as a Molecular Target for the Development of Novel Drugs Against the Dermatophyte *Trichophyton rubrum*. Front. Microbiol..

[86] Martinez-Rossi N.M., Jacob T.R., Sanches P.R., Peres N.T.A., Lang E.A.S., Martins M.P., Rossi A. (2016). Heat Shock Proteins in Dermatophytes: Current Advances and Perspectives. Curr. Genom..

[87] Bitencourt T.A., Neves-da-Rocha J., Martins M.P., Sanches P.R., Lang E.A.S., Bortolossi J.C., Rossi A., Martinez-Rossi N.M. (2021). StuA-regulated processes in the dermatophyte *Trichophyton rubrum*: Transcription profile, cell-cell adhesion, and immunomodulation. Front. Cell. Infect. Microbiol..

[88] Kröber A., Etzrodt S., Bach M., Monod M., Kniemeyer O., Staib P., Brakhage A.A. (2017). The transcriptional regulators SteA and StuA contribute to keratin degradation and sexual reproduction of the dermatophyte *Arthroderma benhamiae*. Curr. Genet..

[89] Liu H., Xu W., Bruno V.M., Phan Q.T., Solis N.V., Woolford C.A., Ehrlich R.L., Shetty A.C., McCraken C., Lin J. (2021). Determining *Aspergillus fumigatus* transcription factor expression and function during invasion of the mammalian lung. PLoS Pathog..

[90] Badali H., Mohammadi R., Mashedi O., de Hoog G.S., Meis J.F. (2015). In vitro susceptibility patterns of clinically important *Trichophyton* and *Epidermophyton* species against nine antifungal drugs. Mycoses.

[91] Salehi Z., Fatahi N., Taran M., Izadi A., Badali H., Hashemi S.J., Rezaie S., Daie Ghazvini R., Ghaffari M., Aala F. (2020). Comparison of in vitro antifungal activity of novel triazoles with available antifungal agents against dermatophyte species caused tinea pedis. J. Mycol. Med..

[92] Chen X., Jiang X., Yang M., Bennett C., González U., Lin X., Hua X., Xue S., Zhang M. (2017). Systemic antifungal therapy for tinea capitis in children: An abridged Cochrane Review. J. Am. Acad. Dermatol..

[93] Tey H.L., Tan A.S.L., Chan Y.C. (2011). Meta-analysis of randomized, controlled trials comparing griseofulvin and terbinafine in the treatment of tinea capitis. J. Am. Acad. Dermatol..

[94] Ebert A., Monod M., Salamin K., Burmester A., Uhrlaß S., Wiegand C., Hipler U., Krüger C., Koch D., Wittig F. (2020). Alarming India-wide phenomenon of antifungal resistance in dermatophytes: A multicentre study. Mycoses.

[95] Martinez-Rossi N.M., Peres N.T.A., Rossi A. (2008). Antifungal Resistance Mechanisms in Dermatophytes. Mycopathologia.

[96] Martins M.P., Rossi A., Sanches P.R., Martinez-Rossi N.M. (2019). Differential expression of multidrug-resistance genes in *Trichophyton rubrum*. J. Integr. OMICS.

[97] Yamada T., Maeda M., Alshahni M.M., Tanaka R., Yaguchi T., Bontems O., Salamin K., Fratti M., Monod M. (2017). Terbinafine Resistance of *Trichophyton* Clinical Isolates Caused by Specific Point Mutations in the Squalene Epoxidase Gene. Antimicrob. Agents Chemother..

[98] Saunte D.M.L., Hare R.K., Jørgensen K.M., Jørgensen R., Deleuran M., Zachariae C.O., Thomsen S.F., Bjørnskov-Halkier L., Kofoed K., Arendrup M.C. (2019). Emerging Terbinafine Resistance in *Trichophyton*: Clinical Characteristics, Squalene Epoxidase Gene Mutations, and a Reliable EUCAST Method for Detection. Antimicrob. Agents Chemother..

[99] Singh A., Masih A., Khurana A., Singh P.K., Gupta M., Hagen F., Meis J.F., Chowdhary A. (2018). High terbinafine resistance in *Trichophyton interdigitale* isolates in Delhi, India harbouring mutations in the squalene epoxidase gene. Mycoses.

[100] Kano R., Kimura U., Kakurai M., Hiruma J., Kamata H., Suga Y., Harada K. (2020). *Trichophyton indotineae* sp. nov.: A New Highly Terbinafine-Resistant Anthropophilic Dermatophyte Species. Mycopathologia.

[101] Tang C., Kong X., Ahmed S., Thakur R., Chowdhary A., Nenoff P., Uhrlass S., Verma S., Meis J., Kandemir H. (2021). Taxonomy of the *Trichophyton mentagrophytes/T. interdigitale* Species Complex Harboring the Highly Virulent, Multiresistant Genotype *T. indotineae*. Mycopathologia.

[102] Monod M., Feuermann M., Salamin K., Fratti M., Makino M., Alshahni M.M., Makimura K., Yamada T. (2019). *Trichophyton rubrum* Azole Resistance Mediated by a New ABC Transporter, TruMDR3. Antimicrob. Agents Chemother..

[103] Cervelatti E.P., Fachin A.L., Ferreira-Nozawa M.S., Martinez-Rossi N.M. (2006). Molecular cloning and characterization of a novel ABC transporter gene in the human pathogen *Trichophyton rubrum*. Med. Mycol..

[104] Martins M.P., Franceschini A.C.C., Jacob T.R., Rossi A., Martinez-Rossi N.M. (2016). Compensatory expression of multidrug-resistance genes encoding ABC transporters in dermatophytes. J. Med. Microbiol..

[105] Fachin A.L., Ferreira-Nozawa M.S., Maccheroni W., Martinez-Rossi N.M. (2006). Role of the ABC transporter TruMDR2 in terbinafine, 4-nitroquinoline N-oxide and ethidium bromide susceptibility in *Trichophyton rubrum*. J. Med. Microbiol..

[106] De Castelo-Branco D.S.C.M., de Aguiar L., Araújo G.D.S., Lopes R.G.P., de Sales J.A., Pereira-Neto W.A., de Pinheiro A.Q., Paixão G.C., de Cordeiro R.A., Sidrim J.J.C. (2020). In vitro and ex vivo biofilms of dermatophytes: A new panorama for the study of antifungal drugs. Biofouling.

[107] Peres N.T.A., Cursino-Santos J.R., Rossi A., Martinez-Rossi N.M. (2011). In vitro susceptibility to antimycotic drug undecanoic acid, a medium-chain fatty acid, is nutrient-dependent in the dermatophyte *Trichophyton rubrum*. World J. Microbiol. Biotechnol..

[108] Lin H., Liu X., Shen Z., Cheng W., Zeng Z., Chen Y., Tang C., Jiang T. (2019). The effect of isoflavaspidic acid PB extracted from *Dryopteris fragrans* (L.) Schott on planktonic and biofilm growth of dermatophytes and the possible mechanism of antibiofilm. J. Ethnopharmacol..

[109] Deng S., Zhang C., Seyedmousavi S., Zhu S., Tan X., Wen Y., Huang X., Lei W., Zhou Z., Fang W. (2015). Comparison of the in vitro activities of newer triazoles and established antifungal agents against *Trichophyton rubrum*. Antimicrob. Agents Chemother..

[110] Hur M.S., Park M., Jung W.H., Lee Y.W. (2019). Evaluation of drug susceptibility test for Efinaconazole compared with conventional antifungal agents. Mycoses.

[111] Roemer T., Krysan D.J. (2014). Antifungal drug development: Challenges, unmet clinical needs, and new approaches. Cold Spring Harb. Perspect. Med..

[112] Yamada T., Makimura K., Abe S. (2006). Isolation, characterization, and disruption of *dnr1*, the *areA/nit-2*-like nitrogen regulatory gene of the zoophilic dermatophyte, *Microsporum canis*. Med. Mycol..

[113] Yamada T., Makimura K., Satoh K., Umeda Y., Ishihara Y., Abe S. (2009). *Agrobacterium tumefaciens*-mediated transformation of the dermatophyte, *Trichophyton mentagrophytes*: An efficient tool for gene transfer. Med. Mycol..

[114] Tudzynski B. (2014). Nitrogen regulation of fungal secondary metabolism in fungi. Front. Microbiol..

[115] Gomes E., Bortolossi J., Sanches P., Mendes N., Martinez-Rossi N., Rossi A. (2018). STE20/PAKA Protein Kinase Gene Releases an Autoinhibitory Domain through Pre-mRNA Alternative Splicing in the Dermatophyte *Trichophyton rubrum*. Int. J. Mol. Sci..

